# Audiometric Outcomes in Chronic Otitis Media with Mastoid Involvement: A Five-Year Clinical Overview

**DOI:** 10.3390/diagnostics14222546

**Published:** 2024-11-13

**Authors:** Cristina Popescu, Renata Maria Văruț, Mihaela Popescu, Alin Iulian Silviu Popescu, Cristina Elena Singer

**Affiliations:** 1 ENT County Hospital Craiova, Discipline of Anatomy, Department of Anatomy, University of Medicine and Pharmacy, 200349 Craiova, Romania; cristina.popescu@umfcv.ro; 2 Research Methodology Department, Faculty of Pharmacy, University of Medicine and Pharmacy of Craiova, 200349 Craiova, Romania; 3 Department of Endocrinology, University of Medicine and Pharmacy of Craiova, 200349 Craiova, Romania; 4 Department of Internal Medicine, University of Medicine and Pharmacy of Craiova, 200349 Craiova, Romania; alin.popescu@umfcv.ro; 5 Department of Mother and Baby, University of Medicine and Pharmacy of Craiova, 200349 Craiova, Romania; cristina.singer@umfcv.ro

**Keywords:** otomastoiditis, hearing loss, audiometric assessment, mixed hearing loss, conductive hearing loss

## Abstract

**Background/Objectives.** Otomastoiditis, an inflammatory condition affecting the middle ear and mastoid cells, poses significant risks for hearing impairment. This study aimed to analyze the clinical presentations, anatomical variations, and audiometric outcomes associated with acute and chronic otomastoiditis over a five-year period at the ENT Clinic of the Clinical County Emergency Hospital of Craiova. **Methods.** A retrospective clinical–statistical analysis was conducted on 145 patients aged 2 to 78 years, who were treated for otomastoiditis. The study involved a comprehensive review of clinical and audiometric data, with a focus on the type of hearing loss (conductive or mixed), audiometric thresholds, and the relationship between the anatomical form of the disease and the severity of hearing loss. **Results.** The majority of cases (93.83%) were chronic otomastoiditis, with 66.89% of patients presenting with mixed hearing loss and 33.10% with conductive hearing loss. Audiometric assessments revealed significant air conduction deficits, particularly at low and mid-range frequencies, with losses averaging 50–55 dB in cases of conductive hearing loss. Chronic cases demonstrated notable bone conduction impairments, indicating progressive cochlear damage. Statistical analysis identified a moderate correlation between the anatomical form of the disease and the severity of hearing loss, particularly in patients with cholesteatomatous-suppurative forms. **Conclusions.** This study underlines the critical need for the early and precise diagnosis of otomastoiditis, supported by audiometric evaluations. Our findings emphasize the substantial risk of progressive cochlear damage in chronic cases, underscoring the necessity for timely intervention to mitigate long-term hearing loss. These results offer valuable insights for clinicians, potentially guiding improved therapeutic approaches and contributing to enhanced patient outcomes in managing chronic otomastoiditis.

## 1. Introduction

Globally, otic pathology ranks among the leading conditions in terms of incidence, associated costs, and impact on quality of life. Consequently, there is a multitude of studies focused on exploring the anatomo-clinical forms of the disease, as well as new diagnostic and treatment methods. Otomastoiditis is an inflammatory process involving the middle ear and mastoid cells [1]. Otic pathology remains one of the most prevalent and costly health issues worldwide, with a profound impact on quality of life due to its association with long-term hearing impairment and its sequelae. Chronic otomastoiditis, in particular, is a significant clinical concern due to its potential to cause progressive and irreversible hearing loss, especially when complicated by cholesteatoma or recurrent infections [2]. Otomastoid involvement often occurs in association with chronic otitis media, particularly in cases complicated by cholesteatoma or when chronic infections are inadequately managed. Despite advances in diagnostic imaging and surgical treatments, managing chronic otomastoiditis remains challenging, especially in complex cases with cholesteatomatous involvement. Given the risk of progressive hearing loss and the challenges in early detection, this study aims to provide detailed audiometric data that could help clinicians identify patterns associated with cochlear damage. Further research is needed to develop effective early intervention strategies that may ultimately improve prognostic outcomes in patients with chronic otomastoiditis [3,4]. Audiometric assessment is essential in managing chronic otomastoiditis, particularly for identifying early signs of cochlear damage that may not be apparent in routine clinical examination. The early detection of audiometric changes allows clinicians to monitor disease progression and adjust therapeutic interventions accordingly, which can potentially minimize long-term hearing impairment. While many studies have investigated general aspects of otitis media, there is limited research specifically exploring audiometric patterns in chronic otomastoiditis. Few studies provide detailed data on how disease progression affects hearing outcomes, particularly in patients with complex cases such as those involving cholesteatoma. This study aims to fill this gap by offering a comprehensive analysis of audiometric changes, contributing to a better understanding of hearing outcomes in chronic otomastoiditis [5,6]. The introduction of antibiotics in the treatment of otitis media has reduced the incidence of otomastoiditis as a complication of acute otitis media from 50% to 0.4%. Recently, several publications have highlighted an increase in the incidence of acute mastoiditis, suggesting a potential correlation between the incorrect use of antibiotic therapy and the rise in cases of mastoiditis [7,8].

The eradication of otomastoid pathological foci through appropriate surgical techniques leads to both the cessation of suppuration and the prevention of complications. If left untreated, otomastoiditis can result in loco-regional complications similar to those of suppurative otitis, including facial paralysis, labyrinthitis, sinuous-jugular thrombophlebitis, otogenic meningitis, epidural or cerebral abscesses, and cerebellar abscesses [9].

Hearing loss represents a particular issue within the broader spectrum of sensory impairments, distinguished by the direct consequences that auditory deprivation has on language and cognitive development, as well as on overall psycho-somatic growth. Specialized research has demonstrated the validity of the concept of ‘auditory deprivation’ and its irreversible effects on the potential for auditory-verbal rehabilitation. It is well-known that the duration of auditory deprivation in a hearing-impaired patient decisively influences their auditory–verbal performance even after prosthetic ‘correction’ of the hearing deficit. Severe, permanent hearing loss that is not detected early will have grave repercussions on speech development, language acquisition, and cognitive development, which in turn will negatively impact emotional and social quality of life. Beyond its negative impact on interpersonal communication, severe bilateral hearing loss also affects other areas of development, including educational attainment, mental health, self-esteem, and employment opportunities [10]. There is a multitude of studies investigating the anatomo-clinical forms of otomastoiditis, with a particular focus on new diagnostic and treatment methods. Chronic suppurative otitis media (CSOM) presents a significant challenge in otorhinolaryngology. The morbidity rate associated with CSOM accounts for 23–30% of ear, nose, and throat (ENT) pathologies, affecting 1–4% of the global population [11].

In recent years, the physiology of the auditory analyzer and its examination methods have been enhanced with new data, allowing for a more precise assessment of its functionality. The restoration and improvement of hearing in patients with chronic suppurative otitis media and its complications remains one of the most challenging issues in otorhinolaryngology today. The accurate diagnosis of otomastoiditis, based on anamnesis, local, and general clinical examination, is complemented by auditory function testing, along with imaging and laboratory examinations [12].

The use of antibiotics as self-medication or in incomplete courses has led to the development of a new pathology by transforming acute otitis media into latent otitis media with an insidious progression, which promotes the occurrence of complications. A hearing examination is crucial for diagnosis, as it reveals conductive hearing loss in the affected ear, characterized by an air conduction loss of 50–60 dB on the tonal audiogram, while bone conduction remains intact; Rinné test negative in the affected ear, prolonged Schwabach, and Weber test lateralized to the affected side. The worsening of hearing loss or cochlear involvement is an indicator of severity. Chronic otomastoiditis with progressive hearing loss has significant implications for patients’ quality of life, affecting communication, social interactions, and overall well-being. Understanding the audiometric impact of this condition is therefore crucial not only for clinical management but also for improving patient outcomes and quality of life.

The objective of this study is to evaluate the audiometric impact of chronic otomastoiditis with mastoid involvement, aiming to correlate clinical presentation and disease severity with hearing outcomes. By identifying specific audiometric patterns, we aim to support otologists in predicting prognosis and tailoring treatment strategies for patients with chronic mastoid pathology.

## 2. Materials and Methods

### 2.1. Study Design

The study is a retrospective clinical–statistical analysis conducted over a period of 5 years (2018–2023) on a cohort of 145 patients (aged 2–78 years) with acute and chronic otic pathology, who were admitted to the ENT Clinic of the Clinical County Emergency Hospital of Craiova. The study cohort was selected from the total number of patients admitted to the clinic. The elements necessary for establishing the ENT diagnosis were obtained by consulting the anamnesis, physical ENT examination, functional ENT examination, general clinical examination, and paraclinical investigations recorded in the patients’ observation sheets. Temporal bone computed tomography (CT) was routinely employed to diagnose otomastoiditis in our cohort using a Siemens CT scanner (Siemens Healthineers, Erlangen, Germany). CT imaging provides detailed visualization of the mastoid air cells, allowing us to detect signs of inflammation, such as fluid accumulation, osteolysis, and destruction of bony septa. In chronic cases, CT also identifies osteocondensations and geodes, which are indicative of long-standing inflammation and bone involvement. In cases requiring surgical intervention (such as mastoidectomy), the diagnosis of otomastoiditis was further confirmed intraoperatively. MRI investigations were performed using the Siemens Magnetom Avanto 1.5T MRI scanner (Siemens Healthineers, Erlangen, Germany). During surgery, direct observation of the mastoid bone frequently revealed signs of active inflammation, including the presence of granulation tissue, osteonecrosis, and in some instances, sequestration of necrotic bone.

### 2.2. Audiometric Examination

Our retrospective study assessed the auditory function in patients diagnosed with otomastoiditis using a combination of tuning fork tests (instrumental acumetry) and pure-tone audiometry. Audiometric testing was conducted using the Sibelsound 400 audiometer (Sibelmed, Barcelona, Spain). These methods were employed to evaluate both air and bone conduction hearing thresholds, allowing for a comprehensive understanding of the auditory impact of the condition. First, tuning fork tests were conducted to explore bone and air conduction hearing. The Schwabach test was utilized to measure the duration of bone conduction. In this procedure, a vibrating tuning fork, calibrated between 128 and 4096 Hz, was placed on the mastoid process. The patient’s perception of sound duration was then compared to standard values, with prolonged bone conduction suggesting conductive hearing loss. Following the Schwabach test, the Rinné test was performed. This test compares air conduction (AC) to bone conduction (BC) by first placing the tuning fork on the mastoid bone and then near the ear canal. A negative Rinné test, in which BC is perceived as louder or longer than AC, is indicative of conductive hearing loss. Finally, the Weber test was carried out to assess sound lateralization. The vibrating tuning fork was placed on the midline of the skull, usually on the vertex, and the patient was asked to indicate which ear heard the sound more prominently. Lateralization to the affected ear often indicates conductive hearing loss, while lateralization to the unaffected ear can suggest sensorineural hearing loss. In cases where all three tests—Weber lateralized to the affected ear, prolonged Schwabach, and negative Rinné—were positive, this combination formed the Bezold’s triad, which is pathognomonic for conductive hearing loss. In addition to the tuning fork tests, pure-tone audiometry was performed in a soundproof environment to assess hearing thresholds more precisely. Frequencies between 125 Hz and 8000 Hz were used, and tones were presented through headphones for air conduction and through a bone vibrator for bone conduction. The intensity of the tones was varied in 5 dB steps. The hearing threshold, defined as the lowest intensity at which the patient could detect sound, was recorded for each frequency. For bone conduction testing, masking was applied to the untested ear if there was a significant difference in hearing acuity (greater than 60 dB) between the ears, to ensure accurate results. Audiometric thresholds were determined using both ascending and descending methods: in the ascending method, testing began at 0 dB, and the intensity was increased until the sound was detected; in the descending method, testing started at a clearly audible level, and the intensity was gradually decreased until the patient could no longer hear the sound. Results from both air and bone conduction tests were plotted on audiograms, with frequencies on the x-axis and sound intensity on the y-axis, allowing us to identify characteristic patterns of hearing loss (conductive, sensorineural, or mixed) [13].

### 2.3. Statistical Analysis

The statistical data were processed using Microsoft Office 2007 for Windows and Microsoft Excel 2007 for Windows; the obtained data were tabulated, with variability represented through graphs. The statistical interpretation of the data was conducted using Chi-square and Cramer’s tests.

The Chi-square test was employed to interpret the contingency tables; the data were evaluated in terms of the dependency between the two classification factors (O—observed frequency, E—expected frequency), considering only the results under 5%, which is regarded as a sufficient significance threshold.

In the Chi-square test for testing the dependency between two factors, the test result was calculated for the data in the contingency tables, and this result was compared to the threshold value indicating a significant dependency (threshold of 95% or 99%) or a highly significant dependency (threshold of 99.9%) between the two classification factors. The following interpretation of *p*-values, provided directly by the software used for statistical data processing, was applied using the above-mentioned test.

- *p* < 0.05: The difference between the two means is significant (S)—95% confidence.
- *p* < 0.01: The difference between the two means is significant (S)—99% confidence.
- *p* < 0.001: The difference between the two means is highly significant (HS)—99.9% confidence.
- *p* > 0.05: The difference between the two means is not significant (NS).

The test shows whether there is any relationship (mutual influence) between two factors. Cramer’s test checks the strength of the association between two nominal factors and is used for tables with multiple rows and columns (for 2 × 2 tables, the phi coefficient is preferred). More specifically, it measures whether each category of one factor preferentially associates with one of the categories of the other factor. The result of this test is denoted by *V*.

$$V=\sqrt{x^{2}/\min\left(r-1,c-1\right)}$$

where *r* and *c* represent the number of rows and columns in the contingency table being studied.

## 3. Results

The analysis of the mentioned cases highlighted the chronic form of otomastoiditis in 137 cases (94.48%), with an evolution longer than three months, while 8 cases (5.52%) presented the acute form of otomastoiditis, with an evolution ranging from 0 to 3 months (Table 1).

The anatomo-clinical forms of otomastoiditis observed in the study were as follows: 5 patients (3.44%) had simple cholesteatomatous forms, 54 cases (37.27%) had suppurative cholesteatomatous forms, 7 cases (4.82%) had simple polypoid forms, 27 cases (18.62%) had suppurative polypoid forms, 32 cases (22.06%) had simple suppurative forms, and 20 cases (13.79%) had combined suppurative polypoid cholesteatomatous forms (Table 2).

### 3.1. Audiometric Results

#### 3.1.1. Instrumental Acumetry with Tuning Forks

The instrumental acumetry tests used in audiological diagnosis revealed changes in bone or air conduction. The interpretation of these tests showed that 54.16% of patients with conductive hearing loss had a negative Rinné test for the left ear, 39.58% had a negative Rinné test for the right ear, and 6.25% had a negative Rinné test for both ears. Similarly, the Weber test in patients with conductive hearing loss showed the same percentages, consistent with the involvement of the right ear, left ear, or both ears, similar to the Rinné test. In the case of patients with mixed hearing loss, the tuning fork test revealed that 51.54% had a negative Rinné test for the left ear, 46.93% had a negative Rinné test for the right ear, and 2.06% had a negative Rinné test for both ears. The same percentages were observed in the Weber test, with 51.54% of patients showing lateralization to the left, 46.93% showing lateralization to the right, and 2.06% showing an indifferent result (Table 3). The audiogram also played a decisive role in determining the postoperative auditory functional evolution, the necessity of eventual hearing aid fitting, and in assessing the functional status of the opposite ear. Only 15% of patients subsequently received hearing aids within the first postoperative year, in cases with minimal hearing loss in the contralateral ear. The air conduction threshold provides information about disorders of the external and middle ear, indicating that sounds need to be louder to be heard. The result of this investigation is an assessment of the degree of sensitivity loss. In 48 (33.57%) audiometric recordings, we observed conductive hearing loss without neurosensory impairment. This conclusion was drawn from the interpretation of pure tone audiograms, where we observed a drop in the air conduction curve, particularly at low and mid-range frequencies.

#### 3.1.2. Pure Tone Audiometry

**Figure 1:**
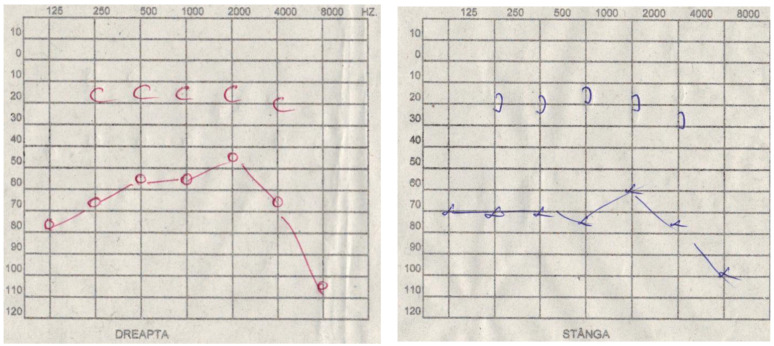
**Right Ear:** Air conduction decreased to 80 dB at 125 Hz, 70 dB at 250 Hz, 55 dB at 500 Hz, 55 dB at 1000 Hz, 45 dB at 2000 Hz, 65 dB at 4000 Hz. **Left Ear:** Air conduction decreased to 70 dB at 125 Hz, 70 dB at 250 Hz, 70 dB at 500 Hz, 75 dB at 1000 Hz, 60 dB at 2000 Hz, 75 dB at 4000 Hz.

A pure tone audiometry was performed on all patients in the cohort (Table 4) and revealed changes in air conduction on the affected side in 48 patients (33.57%), at a frequency of 125 Hz, with hearing loss ranging up to 30 dB in 4 patients (8.33%), 35 dB in 2 patients (4.16%), 40 dB in 6 patients (12.5%), 45 dB in 2 patients (4.16%), 50 dB in 12 patients (25%), 55 dB in 10 patients (20.83%), 60 dB in 3 patients (6.25%), 70 dB in 6 patients (12.5%), and 80 dB in 3 patients (6.25%). At a frequency of 250 Hz, hearing loss ranged up to 30 dB in 2 patients (4.16%), 35 dB in 4 patients (8.33%), 40 dB in 6 patients (12.5%), 45 dB in 6 patients (12.5%), 50 dB in 10 patients (20.83%), 55 dB in 8 patients (16.66%), 60 dB in 6 patients (12.5%), and 70 dB in 4 patients (8.33%). At a frequency of 500 Hz, hearing loss ranged up to 30 dB in 3 patients (6.25%), 35 dB in 6 patients (12.5%), 40 dB in 12 patients (25%), 45 dB in 6 patients (12.5%), 50 dB in 5 patients (10.41%), 55 dB in 4 patients (8.33%), 60 dB in 6 patients (12.5%), and 70 dB in 6 patients (12.5%). At a frequency of 1000 Hz, hearing loss ranged up to 30 dB in 10 patients (20.83%), 35 dB in 4 patients (8.33%), 40 dB in 6 patients (12.5%), 45 dB in 5 patients (10.41%), 50 dB in 4 patients (8.33%), 55 dB in 4 patients (8.33%), 60 dB in 6 patients (12.5%), 65 dB in 3 patients (6.25%), and 75 dB in 6 patients (12.5%) (Figure 1 and Table 5).

**Figure 2:**
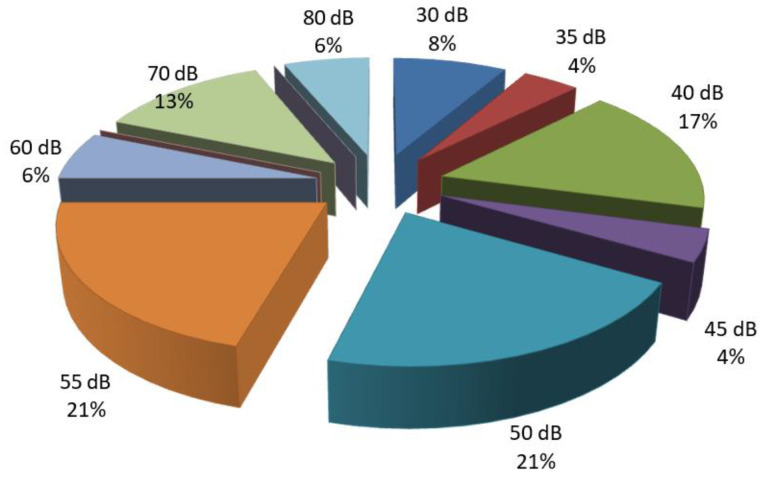
Percentage distribution of patients with conductive hearing loss based on the number of decibels lost at low frequencies.

**Figure 3:**
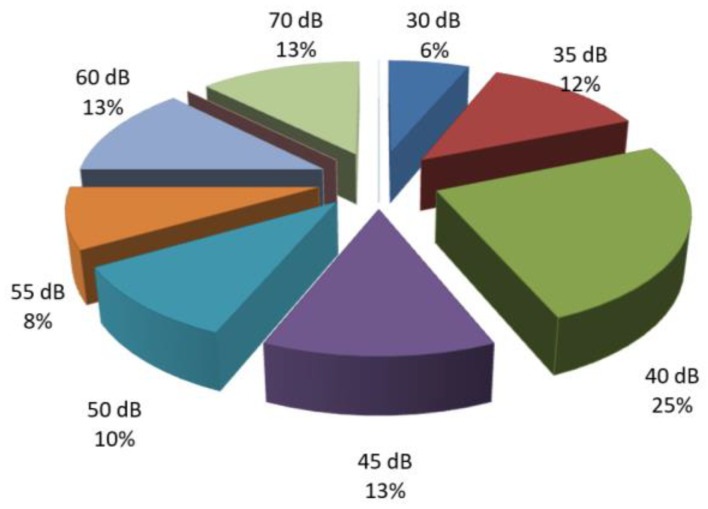
Percentage distribution of patients with conductive hearing loss based on the number of decibels lost at mid frequencies.

Among patients with conductive hearing loss, 21% exhibited a loss of 50–55 dB at low frequencies, while 17% of patients had a loss of 40 dB. Only 6% of patients with conductive hearing loss had a decibel loss of 80 dB (Figure 2 and Figure 3).

In cases with prolonged chronic evolution, bone conduction changes were also observed. Thus, in 97 patients (66.43%), both air and bone conduction were affected. Air conduction decreased at a frequency of 125 Hz, with hearing loss of 30 dB in 5 patients (5.15%), 35 dB in 2 patients (2.06%), 40 dB in 6 patients (6.12%), 45 dB in 16 patients (16.49%), 50 dB in 7 patients (7.14%), 55 dB in 10 patients (10.20%), 60 dB in 3 patients (3.06%), 65 dB in 9 patients (9.18%), 70 dB in 19 patients (19.38%), 75 dB in 8 patients (8.16%), 80 dB in 6 patients (6.12%), and 85 dB in 6 patients (6.12%). At a frequency of 250 Hz, hearing loss ranged up to 30 dB in 4 patients (4.12%), 35 dB in 3 patients (3.09%), 40 dB in 8 patients (8.24%), 45 dB in 9 patients (9.27%), 50 dB in 9 patients (9.27%), 55 dB in 11 patients (11.34%), 60 dB in 7 patients (7.21%), 65 dB in 14 patients (14.43%), 70 dB in 10 patients (10.30%), 75 dB in 10 patients (10.30%), 80 dB in 7 patients (7.21%), and 85 dB in 5 patients (5.15%). At a frequency of 500 Hz, hearing loss ranged up to 35 dB in 5 patients (5.15%), 40 dB in 7 patients (7.21%), 45 dB in 7 patients (7.21%), 50 dB in 12 patients (12.37%), 55 dB in 14 patients (14.43%), 60 dB in 10 patients (10.30%), 65 dB in 9 patients (9.27%), 70 dB in 8 patients (8.24%), 75 dB in 7 patients (7.21%), 80 dB in 11 patients (11.34%), and 85 dB in 5 patients (5.15%). At a frequency of 1000 Hz, hearing loss ranged up to 35 dB in 3 patients (3.09%), 40 dB in 8 patients (8.24%), 45 dB in 5 patients (5.15%), 50 dB in 7 patients (7.21%), 55 dB in 11 patients (11.34%), 60 dB in 17 patients (17.52%), 65 dB in 14 patients (14.43%), 70 dB in 8 patients (8.24%), 75 dB in 10 patients (10.30%), 80 dB in 9 patients (9.27%), and 85 dB in 4 patients (4.12%) (Table 6).

**Figure 4:**
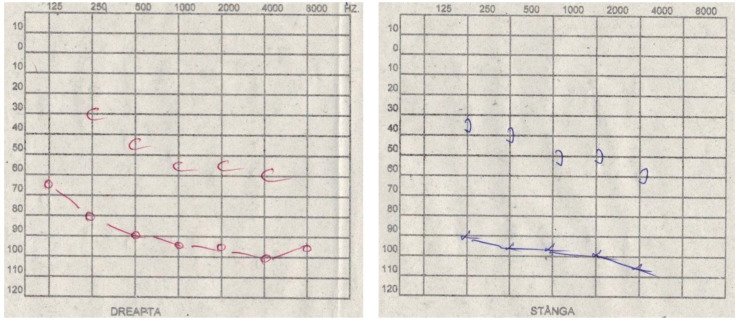
**Right Ear:** Air conduction decreased to 65 dB at 125 Hz, 80 dB at 250 Hz, 90 dB at 500 Hz, 95 dB at 1000 Hz; bone conduction decreased to 30 dB at 250 Hz, 45 dB at 500 Hz, 55 dB at 1000 Hz, 55 dB at 2000 Hz, and 60 dB at 4000 Hz. The gap between bone conduction and air conduction was 50 dB at 250 Hz, 45 dB at 500 Hz, 40 dB at 1000 Hz, 40 dB at 2000 Hz, and 40 dB at 4000 Hz. **Left Ear:** Air conduction decreased to 90 dB at 250 Hz, 95 dB at 500 Hz, 95 dB at 1000 Hz; bone conduction decreased to 35 dB at 250 Hz, 40 dB at 500 Hz, 50 dB at 1000 Hz, 50 dB at 2000 Hz, and 60 dB at 4000 Hz. The gap between bone conduction and air conduction was 55 dB at 250 Hz, 55 dB at 500 Hz, 45 dB at 1000 Hz, 50 dB at 2000 Hz, and 45 dB at 4000 Hz.

Bone conduction was decreased at a frequency of 250 Hz by 10 dB in 8 patients (8.24%), 15 dB in 15 patients (15.46%), 20 dB in 11 patients (11.34%), 25 dB in 7 patients (7.21%), 30 dB in 15 patients (15.46%), 35 dB in 7 patients (7.21%), 40 dB in 10 patients (10.30%), 45 dB in 7 patients (7.21%), 50 dB in 8 patients (8.24%), and 55 dB in 9 patients (9.27%). At a frequency of 500 Hz, it decreased to a loss of 15 dB in 6 patients (6.18%), 20 dB in 19 patients (19.58%), 25 dB in 16 patients (16.49%), 30 dB in 11 patients (11.34%), 35 dB in 15 patients (15.46%), 40 dB in 12 patients (12.37%), 45 dB in 9 patients (9.27%), 50 dB in 7 patients (7.21%), and 55 dB in 3 patients (3.09%). At a frequency of 1000 Hz, it decreased to a loss of 20 dB in 10 patients (10.30%), 25 dB in 13 patients (13.40%), 30 dB in 16 patients (16.49%), 35 dB in 9 patients (9.27%), 40 dB in 17 patients (17.52%), 45 dB in 17 patients (17.52%), 50 dB in 5 patients (5.15%), 55 dB in 5 patients (5.15%), and 60 dB in 5 patients (5.15%). At a frequency of 2000 Hz, it decreased to a loss of 25 dB in 7 patients (7.21%), 30 dB in 9 patients (9.27%), 35 dB in 15 patients (15.46%), 40 dB in 14 patients (14.43%), 45 dB in 15 patients (15.46%), 50 dB in 17 patients (17.52%), 55 dB in 10 patients (10.30%), and 60 dB in 8 patients (8.24%). At a frequency of 4000 Hz, it decreased to a loss of 25 dB in 5 patients (5.15%), 30 dB in 9 patients (9.27%), 35 dB in 11 patients (11.34%), 40 dB in 10 patients (10.30%), 45 dB in 14 patients (14.43%), 50 dB in 17 patients (17.52%), 55 dB in 16 patients (16.49%), 60 dB in 12 patients (12.37%), and 65 dB in 3 patients (3.09%) (Figure 4 and Table 7).

The determination of the bone conduction threshold, which provides information about neurosensory disorders in the inner ear, demonstrated an unaffected curve in cases diagnosed at early stages of the disease, while chronic cases were accompanied by pathological curves that also indicated bone conduction threshold impairment.

**Figure 5:**
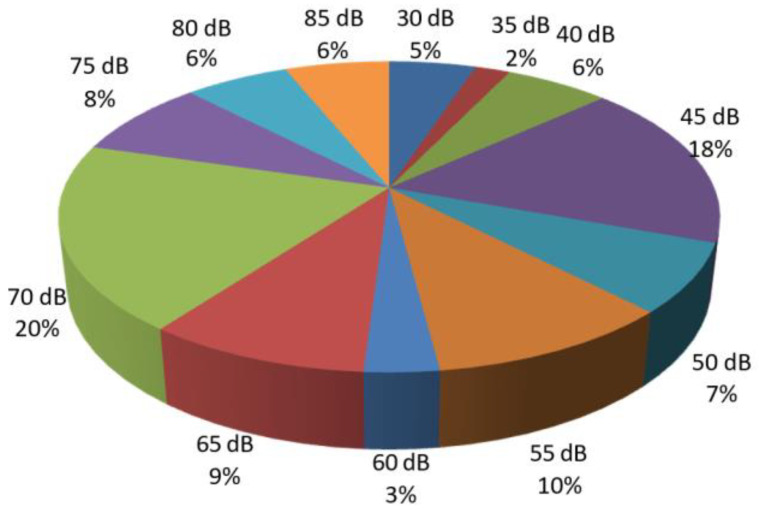
Percentage distribution of patients with mixed hearing loss based on the number of decibels lost via air conduction at low frequencies.

In 97 patients, audiogram analysis revealed mixed hearing loss, with decibel losses present at both low and high frequencies. The following findings were noted: for low frequencies, air conduction loss of 70 dB was the most commonly observed, occurring in 20% of patients, while a similar percentage of 18% exhibited a 45 dB loss at low frequencies (Figure 5).

**Figure 6:**
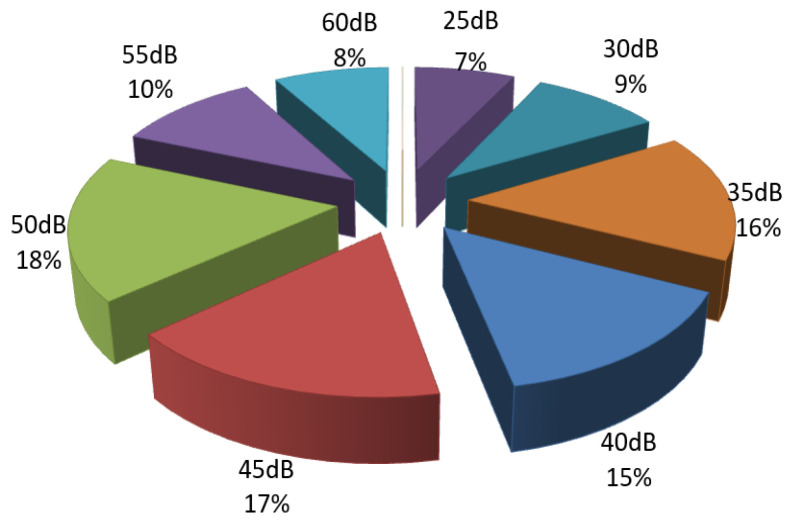
Percentage distribution of patients with mixed hearing loss based on the number of decibels lost via air conduction at high frequencies.

The greatest loss at high frequencies was recorded in 18% of patients with mixed hearing loss, who experienced a 50 dB loss; 16% of patients had a 35 dB loss at high frequencies (Figure 6). These audiometric changes were suggested by the results of the Rinné, Schwabach, and Weber tests. In cases of acute otomastoiditis, a pronounced conductive hearing loss was observed, with an elevated auditory threshold in the affected ear, a negative Rinné test in the affected ear, prolonged Schwabach, and Weber lateralized to the affected side. In chronic cases, tonal audiometry revealed the presence of either conductive or mixed hearing loss; mixed hearing loss indicates a complication with labyrinthine fistula, with cochlear involvement being a factor of poor prognosis. These findings are consistent with numerous specialized studies, which confirm that instrumental acumetry and tonal audiometry are important methods for determining the degree and type of hearing loss, thereby guiding the appropriate therapeutic approach [14].

In the studied group, patients with mixed hearing loss predominated (66.89%), with an average age of 41.56 years, which was higher than that of patients with conductive hearing loss (36.38 years) (Table 8). The prolonged course of aural suppuration and age-related changes in hearing justified the alterations observed in the pure-tone audiogram.

Statistically, the difference between the average age of patients with conductive hearing loss and those with mixed hearing loss was not significant, as indicated by an ANOVA test result of 0.097.

The statistical analysis of the mean deficits recorded at the 125 Hz frequency in patients with mixed hearing loss showed a significant increase (*p* = 0.00518 compared to the 0.05 threshold, indicating statistical significance) relative to the losses described in patients with conductive hearing loss.

For the 250 Hz frequency, a highly significant statistical increase was observed, as evidenced by a *p*-value of 0.00001, which is much lower than the 0.001 threshold.

The association of sensorineural hearing loss with conductive hearing loss in cases of mixed hearing loss led to a highly significant increase in the mean deficits at the 500 Hz and 1000 Hz frequencies in patients with mixed hearing loss compared to those with conductive hearing loss. This statistically significant finding is demonstrated by the recorded *p*-value, which is much lower than the 0.001 threshold.

The same highly significant increase in the mean deficits was also observed at the 2000 Hz and 4000 Hz frequencies in patients with mixed hearing loss compared to those with conductive hearing loss. This finding was statistically demonstrated by the recorded *p*-value (Table 9).

### 3.2. Statistical Analysis Results

#### 3.2.1. Comparison Between the Mean Deficits for Air Conduction Versus Bone Conduction in Patients with Mixed Hearing Loss

In patients with mixed hearing loss, a slight increase in values was observed on the air conduction curve compared to the bone conduction curve at mid and high frequencies. This difference was statistically significant, as indicated by a *p*-value much lower than the 0.001 threshold, which points to a highly significant difference in distribution from a statistical standpoint (Table 10).

#### 3.2.2. Distribution of Patients Based on the Duration of the Condition and Hearing Loss Deficit

The comparative analysis of mean deficits at low frequencies recorded in patients with an otic condition lasting more than 3 months versus those with auricular suppuration lasting less than 3 months yielded a *p*-value of 0.66104 for the 125 Hz frequency and 0.55375 for the 250 Hz frequency. Both values are greater than 0.05, indicating that the difference between the two means is not statistically significant.

*p*-values greater than 0.05, specifically *p* = 0.79121 and *p* = 0.43248, were also observed when comparing the mean hearing deficits at mid frequencies, 500 Hz and 1000 Hz, based on the duration of the disease. Consequently, the difference between the two means was not statistically significant). A slight increase in the mean hearing deficits at 1000 Hz was recorded in patients with an aural condition lasting less than 3 months. However, it must be noted that the small number of cases with a disease duration of less than 3 months did not allow for a satisfactory distribution, which limited the ability to formulate generally applicable hypotheses.

The difference between the two mean hearing deficits at high frequencies, specifically *p* = 0.62643 for 2000 Hz and *p* = 0.76937 for 4000 Hz, was not statistically significant, as both values are above 0.05. A slight increase in the mean value of hearing deficits at 2000 Hz was observed in patients with a disease duration of less than 3 months (Table 11). However, the small number of patients with aural suppuration lasting less than 3 months compared to those with a longer history of otic disease (over 3 months) did not allow for the generalization of the results, as the difference in sample sizes was too large.

#### 3.2.3. Distribution of Patients Based on the Anatomoclinical Form and Audiogram

The hearing loss documented through audiometric examination is the result of the long-term progression of the inflammatory-infectious process within the tympanic cavity and mastoid, a progression that is not influenced by the anatomical–clinical form of the disease. We encountered 5 patients (3.44%) with bilateral epimezotympanic suppuration who underwent surgical intervention five years apart on each ear. The hearing loss of approximately 40 dB in both ears led to communication difficulties, and these patients are currently using hearing aids.

Performing the Chi-square test, we obtained a value of 8.965, which exceeds the 95% confidence threshold, the corresponding *p*-value for this result is 0.03, which is less than 0.05, the maximum threshold indicating statistical significance. Therefore, we can conclude that there is an influence, albeit not very strong, between the type of hearing loss and the anatomoclinical form (Table 12).

The result of the Cramer’s V test, 0.248, indicates a weak association between the categories of the two factors—anatomoclinical form and type of hearing loss.

According to the classification of hearing loss degrees into mild (30–50 dB), moderate (50–70 dB), severe (70–90 dB), and profound (over 90 dB), the following correlations were established between the degrees of hearing loss and the anatomical–clinical form of otomastoiditis.

Performing the Chi-square test yielded a value of 7.442, which is lower than the critical value of 12.59 at the 95% confidence level. Therefore, there is no significant influence between the anatomical–clinical form and the level of hearing loss in patients with conductive hearing loss. The corresponding *p*-value for this result is 0.282, which is statistically insignificant, exceeding the 0.05 threshold for statistical significance.

The result obtained from the Cramér’s V test, 0.278, indicates a weak association between the categories of the two factors.

Performing the Chi-square test yielded a value of 25.397, which, for the 4 × 3 contingency tables, exceeds the critical values at the 95% (12.59), 99% (16.81), and 99.9% (22.46) confidence levels (as shown in Table 13). The corresponding *p*-value for this result is much smaller than 0.001, indicating a very high level of statistical significance. Therefore, we can conclude that there is a strong influence of the anatomical–clinical form on the level of hearing loss in patients with mixed hearing loss.

The result of the Cramér’s V test, 0.360, indicates a considerable association between the categories of the two factors.

## 4. Discussion

In recent years, numerous studies have explored the relationship between otomastoiditis and hearing loss, offering insights that closely align with the findings of our study. One study investigated the audiometric outcomes in patients with cholesteatomatous otomastoiditis, revealing that these patients often experience severe mixed hearing loss due to both ossicular damage and cochlear involvement [15]. This finding resonates with our results, where patients with cholesteatomatous forms exhibited significant hearing deficits, particularly at higher frequencies.

Another comparative study focused on the surgical management of different forms of otomastoiditis. The study concluded that while surgery generally stabilizes hearing in polypoid and suppurative forms, cholesteatomatous forms often result in persistent mixed hearing loss despite intervention [16]. Our study similarly found that patients with cholesteatomatous-suppurative forms had poor postoperative audiometric outcomes, reinforcing the need for early and aggressive treatment.

A study exploring the impact of chronic otomastoiditis on bone conduction demonstrated that chronicity is associated with more severe bone conduction deficits, particularly in frequencies above 1000 Hz [17]. This is consistent with our findings, where chronic cases showed significant impairments in bone conduction thresholds.

Another study evaluated the diagnostic accuracy of tuning fork tests and pure tone audiometry in detecting hearing loss in otomastoiditis patients. Their study confirmed the reliability of these tests in identifying significant air-bone gaps, which is also supported by our use of Rinné and Weber tests to confirm mixed hearing loss in our cohort [18].

Research focusing on the audiometric profiles of patients with combined cholesteatomatous-polypoid-suppurative forms of otomastoiditis reported that these patients were more likely to develop profound mixed hearing loss, a conclusion that aligns with our findings, where combined forms were associated with the highest mean deficits across all frequencies [19].

Furthermore, a study on the correlation between the duration of otomastoiditis and hearing loss severity found that prolonged disease duration significantly increases the risk of severe mixed hearing loss [20]. This observation is mirrored in our study, particularly in patients with a disease history exceeding three months.

An investigation compared hearing outcomes post-surgery in patients with different forms of otomastoiditis. The study concluded that while most forms showed improvement, cholesteatomatous forms often led to persistent deficits, underscoring the aggressive nature of this variant [5]. Our study similarly noted limited improvement in hearing post-surgery in cholesteatomatous cases.

A study exploring the impact of early intervention in otomastoiditis demonstrated that timely surgical intervention significantly reduces the risk of developing severe mixed hearing loss [21]. This finding supports our emphasis on early diagnosis and treatment to prevent irreversible auditory damage.

Research focusing on the relationship between the anatomical extent of otomastoiditis and hearing loss found that more extensive disease correlates with worse audiometric outcomes, particularly in cholesteatomatous cases [22]. This observation is consistent with our results, where extensive disease forms led to the most severe hearing loss.

Lastly, a study analyzed the role of imaging techniques in predicting hearing outcomes in otomastoiditis. They found that detailed imaging could accurately predict the extent of hearing loss, particularly in mixed forms, which aligns with our use of imaging to assess the anatomical impact on auditory function [23].

Our findings contribute to the understanding of audiometric profiles in chronic otitis media with mastoid involvement, especially regarding the extent of sensorineural loss in cholesteatomatous cases. This information is critical for clinicians in tailoring early interventions and improving long-term hearing outcomes. By demonstrating specific audiometric patterns associated with different anatomoclinical forms, our study highlights the importance of regular audiometric assessments for the early detection of cochlear involvement, especially in patients with cholesteatoma, where mixed hearing loss indicates a poorer prognosis. Consistent with recent studies, our findings confirm that prolonged chronicity in otitis media with mastoid involvement significantly affects audiometric outcomes, particularly in high frequencies. Such insights are essential for otologists when considering surgical intervention and long-term management strategies for patients at risk of progressive hearing loss. Future studies with larger cohorts and longitudinal follow-ups could further elucidate the progression patterns observed in chronic otitis media with mastoid involvement, providing otologists with enhanced predictive tools for hearing loss outcomes.

Our study has several limitations that should be acknowledged. Firstly, the retrospective design restricts our ability to establish causal relationships between disease progression and audiometric outcomes. Additionally, the study cohort was limited to patients treated at a single institution, which may affect the generalizability of the findings to other populations. Another limitation is the relatively small sample size, particularly for some of the specific anatomoclinical subtypes of otomastoiditis, which could limit the statistical power of certain subgroup analyses. Finally, while our audiometric assessments provide valuable insights, future studies with longitudinal designs and larger, multi-center cohorts are needed to confirm our findings and to better understand the progression patterns in chronic otomastoiditis.

## 5. Conclusions

Our study provides valuable insights into the audiometric profiles associated with chronic otitis media with mastoid involvement, emphasizing both the frequency-specific hearing loss patterns and the progressive cochlear damage seen in chronic cases. Notably, this research fills a gap in the literature by detailing the audiometric characteristics of different anatomoclinical forms of otomastoiditis, particularly cholesteatomatous and polypoid forms, which are less frequently analyzed in current studies. The identification of mixed hearing loss patterns, especially in patients with prolonged disease duration, underscores the necessity for clinicians to monitor audiometric changes as early indicators of cochlear involvement. These findings are clinically significant as they support the integration of regular audiometric assessments in the management protocol for chronic otomastoiditis patients. By establishing a correlation between specific disease forms and the severity of hearing impairment, our study offers a framework that can assist otologists in predicting hearing prognosis and tailoring intervention strategies to prevent further auditory deterioration. The implications of early intervention highlighted by our data contribute to improved patient outcomes, potentially minimizing long-term auditory deficits and enhancing quality of life.

## Figures and Tables

**Table 1:** Distribution of patients with otomastoiditis based on the type of condition.

Type of Condition	Number of Cases	Percentage
Acute	8	5.52%
Chronic	137	94.48%

**Table 2:** Distribution of patients with otomastoiditis based on anatomoclinical forms.

Anatomo-Clinical Forms	Number of Cases	Percentage
Simple cholesteatomatous	5	3.44%
Suppurative cholesteatomatous	54	37.27%
Simple polypoid	7	4.82%
Suppurative polypoid	27	18.62%
Simple suppurative	32	22.06%
Suppurative polypoid cholesteatomatous	20	13.79%

**Table 3:** Results of the Rinné and Weber Test in Patients with Conductive and Mixed Hearing Loss.

Tuning Fork Tests		Conductive Hearing Loss		Mixed Hearing Loss	
Rinné Test Results	Weber Test Results	Number of Patients	Percentage	Number of Patients	Percentage
Rinné negative left ear	Weber lateralized to the left	26	54.16%	50	51.54%
Rinné negative right ear	Weber lateralized to the right	19	39.58%	45	46.39%
Rinné negative both ears	Weber indifferent	3	6.25%	2	2.06%

**Table 4:** Type of Hearing Loss.

Type of Hearing Loss	Number of Patients	Percentage
Conductive hearing loss	48	33.57%
Mixed hearing loss	97	66.43%

**Table 5:** Comparative Distribution by Frequency Categories of Air Conduction Hearing Loss in Patients with Conductive Hearing Loss (Number of Patients).

	125 Hz	250 Hz	500 Hz	1000 Hz	2000 Hz	4000 Hz
30 dB	4	2	3	10	6	4
35 dB	2	4	6	4	8	6
40 dB	6	6	12	6	6	12
45 dB	2	6	6	5	3	6
50 dB	12	10	5	4	9	-
55 dB	10	8	4	4	5	3
60 dB	3	6	6	6	5	8
65 dB	-	-	-	3	-	6
70 dB	6	4	6	-	2	3
75 dB	-	-	-	6	4	-
80 dB	3	-	-	-	-	-

**Table 6:** Comparative Distribution by Frequency Categories of Air Conduction Hearing Loss in Patients with Mixed Hearing Loss (Number of Patients).

	125 Hz	250 Hz	500 Hz	1000 Hz	2000 Hz	4000 Hz
30 dB	5	4	-	-	-	-
35 dB	2	3	5	3	-	-
40 dB	6	8	7	8	6	-
45 dB	16	9	7	5	7	-
50 dB	7	9	12	7	6	9
55 dB	10	11	14	11	7	5
60 dB	3	7	10	17	15	7
65 dB	9	14	9	14	13	9
70 dB	19	10	8	8	10	17
75 dB	8	10	7	10	14	24
80 dB	6	7	11	9	12	16
85 dB	6	5	5	4	7	11

**Table 7:** Comparative Distribution by Frequency Categories of Bone Conduction Hearing Loss in Patients with Mixed Hearing Loss (Number of Patients).

	250 Hz	500 Hz	1000 Hz	2000 Hz	4000 Hz
10 dB	8	-	-	-	-
15 dB	15	6	-	-	-
20 dB	11	19	10	-	-
25 dB	7	16	13	7	5
30 dB	15	11	16	9	9
35 dB	7	15	9	15	11
40 dB	10	12	17	14	10
45 dB	7	8	17	15	14
50 dB	8	7	5	17	17
55 dB	9	3	5	10	16
60 dB	-	-	5	8	12
65 dB	-	-	-	-	3

**Table 8:** Comparison of the Mean Age of Patients by Type of Hearing Loss.

Statistics	Total	Mixed Hearing Loss	Conductive Hearing Loss
Number of Patients	145	97	48
Percentage	100.00	66.89	33.10
Mean	39.86	41.56	36.38
Standard Deviation	17.74	17.00	18.87
C.V. (%)	44.36%	40.70%	51.33%

**Table 9:** Comparison of Mean Deficits at 125 Hz, 250 Hz, 500 Hz,1000 Hz, 2000 Hz, and 4000 Hz. Frequencies Between Patients with Conductive Hearing Loss (T) and Those with Mixed Hearing Loss (M).

Frequency/Metric	Number of Patients	Mean (dB)	Standard Deviation (dB)	C.V. (%)
125 Hz	145	56.99	15.0	26.23
125 Hz M	97	59.29	15.38	25.81
125 Hz T	48	52.29	13.13	24.84
250 Hz	145	55.92	14.54	25.91
250 Hz M	97	59.34	14.92	25.01
250 Hz T	48	48.96	14.65	29.67
500 Hz	145	56.37	14.98	26.48
500 Hz M	97	60.51	14.58	23.97
500 Hz T	48	47.92	12.02	24.82
1000 Hz	145	57.29	15.18	26.40
1000 Hz M	97	61.58	13.35	21.56
1000 Hz T	48	48.54	15.05	30.68
2000 Hz	145	59.14	15.34	25.85
2000 Hz M	97	64.64	13.05	20.08
2000 Hz T	48	47.92	13.52	27.92
4000 Hz	145	63.42	15.30	24.04
4000 Hz M	97	70.77	10.29	14.46
4000 Hz T	48	48.44	12.72	25.99

**Table 10:** Mean deficits in patients with mixed hearing loss, air conduction vs. bone conduction.

250 Hz			500 Hz		1000 Hz		2000 Hz		4000 Hz	
Statistics	Air	Bone	Air	Bone	Air	Bone	Air	Bone	Air	Bone
Number of patients	97	97	97	97	97	97	97	97	97	97
Mean	59.34	31.12	60.51	31.89	61.58	37.09	64.64	43.27	70.77	45.92
Standard deviation	14.92	14.19	14.58	10.92	13.35	11.12	13.05	10.05	10.29	10.75
C.V. (%)	25.0%	45.36%	23.9%	34.08%	21.5%	29.83%	20.1%	23.1%	14.4%	23.30%

**Table 11:** Distribution of patients based on the duration of the condition and hearing loss deficit at low and mid frequencies.

Frequency/Metric	Number of Patients	Mean (dB)	Standard Deviation (dB)	C.V. (%)
125 Hz	145	56.99	15.00	26.23
125 Hz > 3 months	138	57.10	15.16	26.45
125 Hz < 3 months	7	55.00	12.54	21.32
250 Hz	145	55.93	14.54	25.91
250 Hz > 3 months	138	56.05	14.78	26.27
250 Hz < 3 months	7	53.75	9.91	17.25
500 Hz	145	56.37	14.98	26.48
500 Hz > 3 months	138	56.45	15.07	26.60
500 Hz < 3 months	7	55.00	14.14	24.05
1000 Hz	145	57.30	15.18	26.40
1000 Hz > 3 months	138	57.10	15.38	26.83
1000 Hz < 3 months	7	60.63	11.48	17.71
2000 Hz	145	59.14	15.34	25.85
2000 Hz > 3 months	138	59.02	15.54	26.24
2000 Hz < 3 months	7	61.25	11.88	18.14
4000 Hz	145	63.43	15.30	24.04
4000 Hz > 3 months	138	63.51	15.41	24.17
4000 Hz < 3 months	7	61.88	14.13	21.36

**Table 12:** Relationship between the anatomoclinical form and the type of hearing loss.

Type of Hearing Loss	Cholesteatomatous Forms	Polypoid Forms	Suppurative Forms	Cholesteatomatous-Polypoid-Suppurative Forms	Total
Conductive	17	15	14	2	48
Mixed	43	18	18	18	97
Total	60	33	32	20	145

**Table 13:** Relationship between the anatomoclinical form and the degrees of hearing loss in patients with conductive and mixed hearing loss.

Conductive Hearing Loss						Mixed Hearing Loss				
Degrees of Conductive Hearing Loss	Cholesteatomatous Form	Polypoid Form	Suppurative Form	Cholesteatomatous-Polypoid-Suppurative Form	Total	Cholesteatomatous Form	Polypoid Form	Suppurative Form	Cholesteatomatous-Polypoid-Suppurative Form	Total
HU	11.76%	20.00%	35.71%	0.00%	20.83%	16.28%	10.53%	38.89%	0.00%	16.33%
HM	52.94%	53.33%	42.86%	0.00%	47.92%	51.16%	57.89%	44.44%	16.67%	44.90%
HI	35.29%	26.67%	21.43%	100.00%	31.25%	32.56%	31.58%	16.67%	83.33%	38.78%

HU (Hearing Unaffected), HM (Hearing Mild) HI (Hearing Impaired).

## Data Availability

Data are contained within the article.
